# Utility of CSF in translational neuroscience

**DOI:** 10.1007/s10928-013-9301-9

**Published:** 2013-02-12

**Authors:** Elizabeth C. M. de Lange

**Affiliations:** Leiden-Amsterdam Center for Drug Research, Leiden, The Netherlands

**Keywords:** Target site, Cerebrospinal fluid, Human, Central nervous system, Translational, Physiologically based pharmacokinetic model

## Abstract

Human cerebrospinal fluid (CSF) sampling is of high value as the only general applicable methodology to obtain information on free drug concentrations in individual human brain. As the ultimate interest is in the free drug concentration at the CNS target site, the question is what CSF concentrations may tell us in that respect. Studies have been performed in rats and other animals for which concentrations in brain extracellular fluid (brain ECF) as a target site for many drugs, have been compared to (cisterna magna) CSF concentrations, at presumed steady state conditions,. The data indicated that CSF drug concentrations provided a rather good indication of, but not a reliable measure for predicting brain ECF concentrations. Furthermore, comparing rat with human CSF concentrations, human CSF concentrations tend to be higher and display much more variability. However, this comparison of CSF concentrations cannot be a direct one, as humans probably had a disease for which CSF was collected in the first place, while the rats were healthy. In order to be able to more accurately predict human brain ECF concentrations, understanding of the complexity of the CNS in terms of intrabrain pharmacokinetic relationships and the influence of CNS disorders on brain pharmacokinetics needs to be increased. This can be achieved by expanding a currently existing preclinically derived physiologically based pharmacokinetic model for brain distribution. This model has been shown to successfully predict data obtained for human lumbar CSF concentrations of acetaminophen which renders trust in the model prediction of human brain ECF concentrations. This model should further evolute by inclusion of influences of drug properties, fluid flows, transporter functionalities and different disease conditions. Finally the model should include measures of target site engagement and CNS effects, to ultimately learn about concentrations that best predict particular target site concentrations, via human CSF concentrations.

## Introduction

The treatment of neurological diseases is a huge problem. The search for appropriate treatments is under increased pressure as, on one hand, the results of drug candidates in clinical trials are very disappointing, while on the other hand an increase in the incidence of neurological diseases is occurring, probably due to an ageing society and life styles choices.

While having a significant effect in laboratory species, the failure of central nervous system (CNS) drug candidates in clinical phases may be caused by the drug candidate being given to the wrong subjects, administered at the wrong dose or schedule, or because of the lack of proper detection of the favorable effects of the CNS drug candidate. Thus, reasons for failure of CNS drug candidates can at least in part be found in inconclusive pharmacokinetic data, particularly regarding blood brain barrier (BBB) transport, inconclusive pharmacodynamic data, and the variability of the data due to the heterogenous nature of CNS pathologies in humans [1]. Clearly, CNS drug research so far has not yielded solutions and the question is how to have the *right drug, at the right time, at the right concentration, at the right place?* To that end some main issues need to be addressed:

- How to obtain information on (what can be referred to as) the site of action in the CNS?
- How to appropriately diagnose neurological diseases, especially in an early stage?
- How to determine the effect of the drug on treatment of the disease?

To answer the first question, it is important to investigate particular the free CNS drug concentrations, These concentrations may serve as the best predictor of drug effects because CNS drug targets such as receptors, enzymes and transporters only interact with the free drug concentration [2–10]. Emphasis has therefore been put on obtaining reliable estimates of free drug concentrations in the brain extracellular (brain ECF) space that faces many of these targets [11]. The most straightforward method to obtain information on free drug concentrations in human brain ECF is by cerebrospinal fluid (CSF) sampling. CSF is in close contact with brain ECF and therefore is expected to reflect the brain ECF composition. Human CSF sampling has been used (sparsely) for a long time as proof of CNS penetration, for CSF biomarker evaluation, and for pharmacokinetic-pharmacodynamic (PK-PD) modeling of CNS drugs.

However, today, questions abound on the utility of CSF sampling in neuroscience. It is therefore important to critically evaluate the use of CSF drug concentrations as source for information on brain ECF concentrations. The literature provides many, mostly older, studies in which CSF concentrations have been determined, and compared with (total) plasma concentrations, mostly at single time points, under assumed steady state conditions. With the later introduction of the microdialysis technique, information on the relationships between CSF concentrations and brain ECF concentrations became available. Such studies were typically performed in rodents because the brain microdialysis technique is invasive, though minimally [12]. In humans, this technique has only been applied in extreme conditions such as brain trauma and brain surgery. For ethical reasons it has never been used in healthy volunteers (for details see Shannon et al. in this special issue).

This article will provide information on brain compartments from a physiological and anatomical perspective as basis for interpretation of drug transport into and within the brain. Studies will be presented in which CSF and brain ECF concentrations have been assessed and compared, followed by a systematic approach towards that leads to the possibility to use human CSF values to predict target site concentrations of CNS drugs [13].

### CNS compartments and drug transport processes

#### Anatomy and physiology of the CNS

**Fig. 1:**
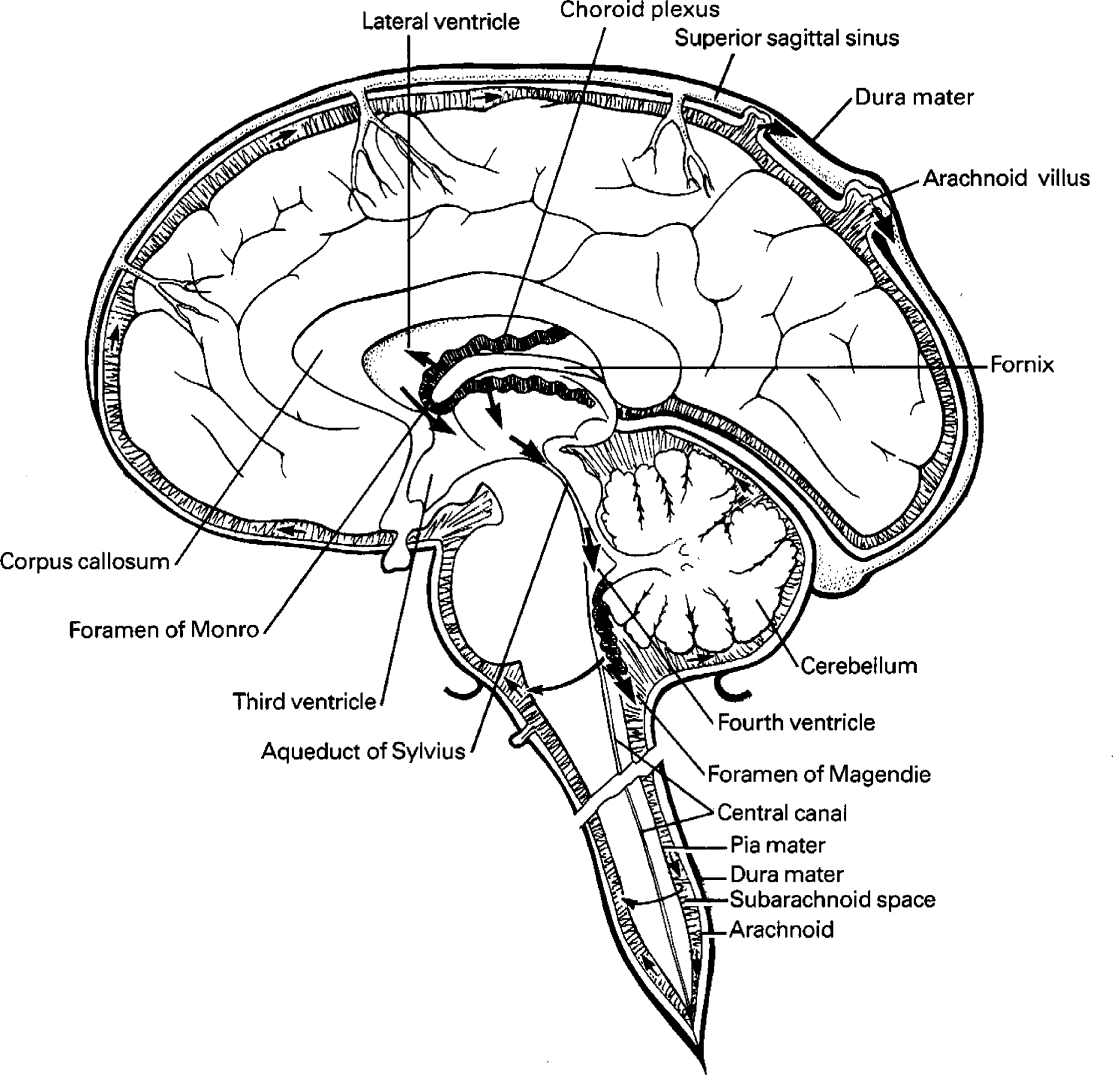
Production, circulation and resorption of cerebrospinal fluid. The production of CSF mostly takes place in the choroid plexus of the ventricles. CSF circulates from the ventricles to the subarachnoid spaces, where resorption takes place via the arachnoid granulations and villi during circulation. From Kandel and Schwartz, Principles of Neural Science, Elsevier Science Publishing, NY, 1985, with permission

The anatomy of the CNS is complex. It can grossly be divided into the following main compartments: brain extracellular fluid (brain ECF), brain parenchyma (brain cells), and the ventricular system. The ventricular system can be seen as a communicating network of cavities filled with CSF. It can be subdivided into the right and left lateral ventricles, the third ventricle, the cerebral aqueduct, the fourth ventricle, the cisterna magna, and the subarachnoid space (Fig. 1). In humans, the subarachnoid CSF in the lumbar region of the spinal cord (lumbar CSF) is of most interest with regard to CSF sampling, because of its accessability.

The exchange of substances from the brain ECF and CSF are regulated by the blood–brain barriers, i.e., the endothelial and epithelial interfaces that serve as barriers to blood. The endothelial cells of the brain capillaries that constitute the blood brain barrier (BBB) have tight junctions in the paracellular space that render this cell layer relatively impermeable to most hydrophilic molecules. In addition a plethora of transport mechanisms are available that control the exchange of compounds across the BBB [14, 15].

The choroid plexus and arachnoidal epithelial cells comprise the blood–CSF barrier (BCSFB). The BCSFB also restricts and highly controlsthe exchange of compounds. The BCSFB is comparable but quantitatively and qualitatively not equal to the BBB [16–18]. More permeable capillaries are found in the circum ventricular organs (CVOs), such as the subfornical organ in the third ventricle region [19], than at the BBB.

**Fig. 2:**
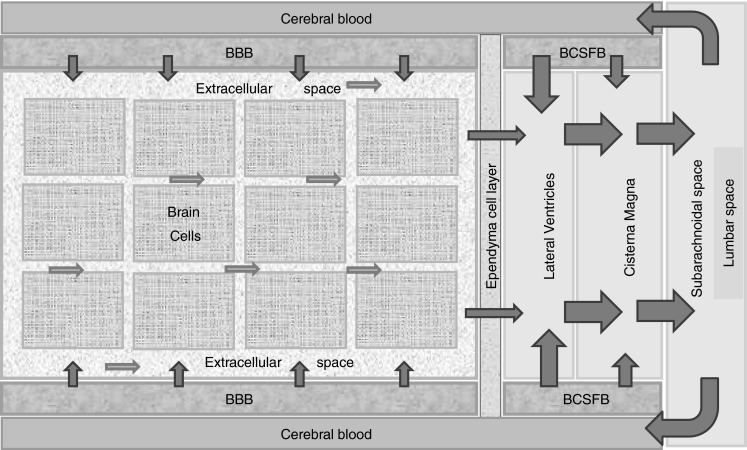
Simplifed and schematic representation of the production, flow and elimination of brain fluids

CSF is continuously produced and eliminated. It provides a continuous circulation that acts more or less like a brain drainage system (Fig. 2). Under normal circumstances the human brain produces ~0.35 ml/min (500 ml/day). The total volume of human CSF is ~150 ml. This indicates that the CSF turnover is ~4 times/day [19]. Although the exact locations of CSF production are still not clear there are known CSF production sites. The majority of CSF is formed in the choroid plexus of the lateral ventricles. Smaller amounts are formed by the choroid plexus of the third and fourth ventricles. The production is mediated by the filtration of plasma through fenestrated capillaries and by active transport of water and dissolved substances through the epithelial cells of the BCSFB. In addition, there are indications that drainage of the brain ECF contributes to CSF formation [15]. Furthermore, it has been demonstrated that a substantial portion of subarachnoid CSF cycles through the brain interstitial space [20, 21]. Elimination of CSF takes place mostly via the granulations (kind of valves) of the arachnoid villi, where CSF flows into the blood. This process is driven by the hydrostatic pressure difference between the CSF and the cerebral veins [22, 23]. In addition, spinal venous reabsorption of CSF has been proven to exist [24].

In the rat these processes are similar although the particular physiological volumes and flow rates have different values. Table 1 shows the values for both rat and human, which is important in scaling by physiologically-based PK modeling [25].

**Table 1:** Human and rat approximate values for brain physiological parameters

Parameter	Human value	Rat value
CSF volume	150 ml [19]	250 ul [65]))
CSF production	0.35 ml/min [19]	2.2 ul/min [66, 67]
CSF turnover	4 times/day [19]	11 times/day [19]
Brain weight	1400 g [68]	1.8 g (own observations)
Brain ECF volume	240 ml [14]	290 ul [66, 67]
Brain ECF production (bulk flow)	0.15–0.20 ml/min [14, 15, 69]	0.2–0.5 ul/min [15, 66]
Cerebral blood flow	700 ml/min [70]	1.1 ml/min [71]

#### Processes that govern local pharmacokinetics of the free drug in the CNS

**Fig. 3:**
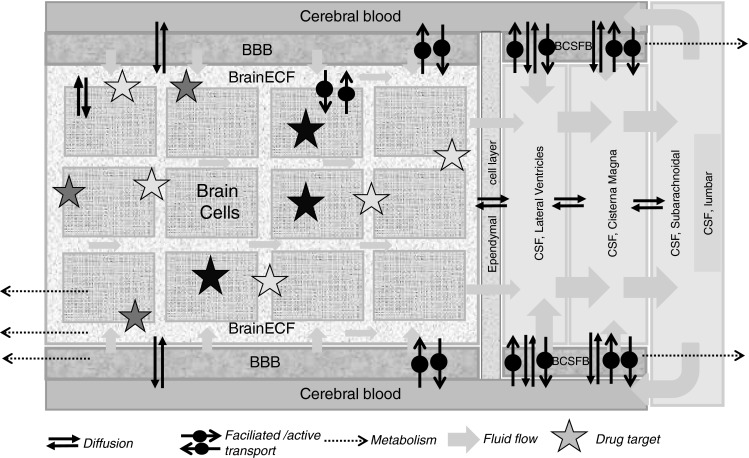
Simplified and schematic representation passive and active transport processes, metabolism, and fluid flow, that all govern the concentration–time profile of the free drug at different sites in the CNS, and therewith CNS drug effects

Drug transport into, within, and out of the brain is governed by the free drug concentrations in plasma, transport across the brain barriers, CSF turnover and ECF bulk flow, extra-intracellular exchange, brain tissue binding and brain drug metabolism (Fig. 3). These processes have been extensively addressed in previous publications [11, 16, 26–28]. It is important to note that transport across the blood–brain barriers may occur by simple diffusion, facilitated diffusion, vesicle transport, or active transport, or combinations of these, depending on the drug. All these processes occur concomitantly, and will influence each other’s rate and extent, such that these interrelationships need to be considered in ultimately predicting CNS target site concentrations, and resulting drug effects [2, 29].

As many important CNS targets currently identified are membrane bound receptors facing the brain ECF, the concentrations in this compartment are of high value in availability of the drug to interact with its CNS target. In order to have the *right drug at the right place at the right time in the right concentrations*, the drug properties and pharmacokinetic processes that govern drug concentrations at specific CNS sites, must be known, ideally with measurements included on target engagement and on drug effects.

## What methods are available to obtain information on unbound brain pharmacokinetic data?

For a long time, monitoring approaches have been searched for that could be used to predict human target site kinetics and (therewith) CNS effects. As it is the free drug that is available for target binding, in the early 1980s it was anticipated that CSF drug concentrations could serve as a biomarker for free brain target site concentrations (with no protein binding of drugs in CSF taking place at least under physiological conditions) [30].

### Single and repeated CSF sampling

The simplest way to study the entry of drugs into the CNS is to measure drug concentrations in the lumbar CSF collected by a single lumbar puncture during continuous intravenous infusion. While CSF may not necessarily be equal to or closely resembles CNS target site concentrations, this method provides information on the extent of drug distribution between plasma and lumbar CSF. This may be useful knowledge, but leaves out any information on time-dependency (rate) [31]. Via a permanent cannula in the cisterna magna, it is possible to perform repeated (serial) CSF sampling in rats. Thus, the time-course of concentrations in CSF can be obtained in parallelly with CNS drug effects. This provides the possibility of within-subject cross-over designed studies [32]. Also in humans sequential repeated CSF sampling is possible [33]. By in repeated CSF sampling, however, the constant withdrawal of CSF may influence physiology. This is a concern and must be taken into account [34].

### Microdialysis

Microdialysis is a technique that is based on the constant perfusion of a very small probe, with a tip that consists of a semipermeable membrane. Molecules small enough to pass this membrane will traverse the semipermeable membrane towards the lowest concentration according to the concentration gradient. When placed into a tissue, molecules from the extracellular space will enter the perfusate, which becomes the dialysate that can be collected outside of the subject, and can subsequently be analyzed on the compound of interest by an analytical technique of choice, to have information on the compound as a function of time in the dialysate. Dialysate concentrations reflect the true unbound PK in the extracellular fluid surrounding of the microdialysis tip, which can be estimated after appropriate correction for so called “in vivo recovery” [35–39].

To date no other technique is able to obtain such quantitative and time resolved information on the unbound drug of interest as by CSF sampling. However, while minimally invasive to the brain of rats [12], the technique is not widely applicable to human brain. Even the smallest injury to the human brain made by choice should be avoided, if it were not for substantial motifs in benefiting the patient otherwise. Thus, microdialysis has been applied frequently in trauma patients. Monitoring concentrations of (e.g.) lactate and pyruvate as biological markers of brain tissue injury in traumatic brain can be very helpful in the judgement of the condition of the patient and to aid in treatment decisions, well before being able to observe and judge this on the basis of clinical assessments [40]. Microdialysis in human brain can also be combined with neurosurgical procedures, for example before removal of epileptogenic brain tissue. In such settings microdialysis can also be used to measure brain penetration of drugs [41–46], and provides the possibility to determine brain pharmacokinetics in conjunction with resulting biochemical efficacy of therapeutic approaches [40, 47, 48].

Because microdialysis, for obvious ethical reasons, cannot be applied as a technique to be used in humans for the purpose of drug development, human CSF is still considered the best possible fluid to obtain from humans that approximates unbound drug concentrations in brain ECF [49]. The question remains how closely CSF concentrations reflect brain ECF concentrations in different locations in the brain, in diseases, and for different drugs.

### Available data on CSF–ECF relationships

With the introduction of microdialysis techniques, the possibility of making a direct comparison of CSF and brain ECF concentrations in animals became available. Also, intrabrain distribution aspects of drugs could be determined. Sawchuk’s group at the University in Minnesota was the first to do so. In rats, zidovudine and stavudine concentration–time profiles were obtained in CSF and Brain ECF, and challenged by inhibition of active transport processes [50–52]. Shen et al. [27] provided an extensive overview on CSF and brain ECF concentrations for drugs from many therapeutic classes with a wide spectrum in physico-chemical properties. Appending data of more recent studies, Table 2 presents CSF -brain ECF relationships for a broad set of drugs, including the methodology of assessment. These values have resulted from microdialysis to obtain brain ECF as well as CSF concentrations, CSF sampling for assessing CSF concentrations, as well as he brain slice method to obtain brain ECF concentrations. The brain slice method estimates brain ECF concentrations by determining total brain concentrations after drug administration, and to correct for the free fraction as obtained in brain slices after bathing the slice in a buffer solution containing the drug(s) of interest [53, 54].

**Table 2:** Drugs and their Kpuu, CSF and Kpuu, brain values obtained in animals (rats, sheep, rabbits, rhesus monkeys, and nonhuman primates) on the basis of steady state (SS) values, AUC values, continuous infusion (cont inf)

Compound	Species	Time	Kpuu, CSF	Kpuu, brain	Reference
9-OH-Risperidone	Rat	6 h cont inf	0,0684	0,0143	[61]
Acetaminophen	Rat	AUC (partial)	0,3	1,2	[25]
Alovudine	Rat	SS	0,4	0,16	[72]
Antipyrine	Rat	2 h cont inf	0,99	0,708	[56]
Atomoxetine	Rat	SS	1,7	0,7	[64]
Baclofen	Rat	Pseudo SS	0,028	0,035	[73]
Benzylpenicillin	Rat	2 h cont inf	0,0134	0,0264	[56]
Buspirone	Rat	2 h cont inf	0,558	0,612	[56]
Caffeine	Rat	2 h cont inf	1,03	0,584	[56]
Carbamazepine	Rat	2 h cont inf	0,535	0,771	[56]
Carbamazepine	Rat	6 h cont inf	0,167	0,232	[61]
Carboplatin	Nonhuman primate	AUC	0,05	0,05	[74]
Cefodizime	Rat	Specific time	0,02	0,22	[75]
Ceftazidime	Rat	SS	0,039	0,022	[76]
Ceftriaxone	Rat	SS	0,71	0,8	[76]
Cephalexin	Rat	2 h cont inf	0,0225	0,016	[56]
Cimetidine	Rat	2 h cont inf	0,0211	0,00981	[56]
Cisplatin	Nonhuman primate	AUC	0,05	0,05	[74]
Citalopram	Rat	2 h cont inf	0,667	0,494	[56]
Citalopram	Rat	6 h cont inf	0,5	0,559	[61]
CP-615,003	Rat	SS	0,01	0,0014	[77]
Daidzein	Rat	2 h cont inf	0,189	0,0667	[56]
Dantrolene	Rat	2 h cont inf	0,0838	0,0297	[56]
Diazepam	Rat	2 h cont inf	0,847	0,805	[56]
Diphenylhydramine	Sheep	SS (adults)	3,4	3,4	[78]
Diphenylhydramine	Sheep	SS (30 d lambs)	4,9	6,6	[78]
Diphenylhydramine	Sheep	SS (10 d lambs)	5,6	6,6	[78]
EAB515	Rat	15 h	0,18	0,08	[51]
Flavopiridol	Rat	2 h cont inf	0,216	0,0525	[56]
Fleroxacin	Rat	2 h cont inf	0,283	0,25	[56]
Fleroxacin	Rat	SS	0,42	0,15	[79]
Ganciclovir	Rat	6 h cont inf	0,0647	0,0711	[61]
Genistein	Rat	2 h cont inf	0,589	0,181	[56]
Lamotrigine	Rat	SS	1,5	1	[62]
Loperamide	Rat	2 h cont inf	0,0376	0,00886	[56]
Metoclopramide	Rat	6 h cont inf	0,169	0,235	[61]
Midazolam	Rat	2 h cont inf	1,35	2,19	[56]
Morphine	Rat	AUC	0,197	0,51	[80]
M6G	Rat	AUC	0,029	0,56	[80]
*N*-desmethylclozapine	Rat	6 h cont inf	0,0151	0,01	[61]
Norfloxacin	Rat	SS	0,033	0,034	[79]
Ofloxacin	Rat	SS	0,23	0,12	[79]
Oxaliplatin	Nonhuman primate	AUC	0,65	5,3	[74]
Perfloxacin	Rat	2 h cont inf	0,389	0,199	[56]
Perfloxacin	Rat	SS	0,37	0,15	[79]
Phenytoin	Rat	2 h cont inf	0,396	0,447	[56]
Phenytoin	Rat	AUC	0,2	0,85	[81]
Probenecid	Rat	SS	0,6	0,2	[82]
Quinidine	Rat	2 h cont inf	0,0911	0,026	[56]
Quinidine	Rat	6 h cont inf	0,0969	0,0459	[61]
Risperidone	Rat	2 h cont inf	0,124	0,0787	[56]
Risperidone	Rat	6 h cont inf	0,0913	0,0422	[61]
Salicylate	Rat	SS	0,5	0,1	[82]
SDZ EAA 494	Rat	AUC	0,17	0,11	[83]
Sertraline	Rat	2 h cont inf	0,832	1,85	[56]
Stavudine	Rat	SS	0,5	0,3	[84]
Stavudine	Rat	SS	0,6	0,6	[52]
Sulpiride	Rat	2 h cont inf	0,0499	0,0219	[56]
Thiopental	Rat	2 h cont inf	0,599	0,911	[56]
Thiopental	Rat	6 h cont inf	0,0663	0,0663	[61]
Tiagabine	Rat	AUC	0,011	0,011	[85]
Verapamil	Rat	2 h cont inf	0,333	0,0786	[56]
YM992	Rat	SS	1,7	1,4	[86]
Zidovudin	Rabbit	AUC	0,17	0,08	[50]
Zidovudin	Rabbit	SS	0,29	0,19	[87]
Zidovudin	Rhesus Monkey	SS	0,27	0,15	[88]
Zidovudin + probenecid	Rabbit	AUC	0,19	0,1	[50]
Zolpidem	Rat	2 h cont inf	0,475	0,447	[56]

**Fig. 4:**
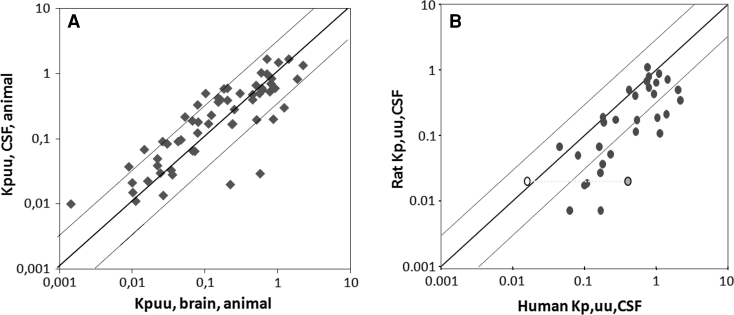
**a** Relationship between the CSF/plasma unbound (Kpuu, CSF) and brain ECF/plasma unbound ratios (Kpuu, brain) obtained at presumed steady state conditions or by AUC comparisons) in healthy animals for data depicted in Table 2, and **b** for rat versus human Kpuu, CSF values (redrawn from Friden et al. (2009)). Note in **b** the *open circle* representing a value for moxalactam obtained from a healthy volunteer, and on the same horizontal line the *half filled circle* representing a value for moxalactam obtained from a patient with bacterial meningitis

As a measure for the extent of distribution, the “Kpuu” value is used and defined as the ratio of the unbound tissue (brain) concentration over the unbound plasma concentration, at equilibrium. Figure 4 shows the relationship of observed data on Kpuu, CSF and Kpuu, brain, obtained from the data presented in Table 2 for healthy rats, as well as the relationship between rat and human Kpuu, CSF values [55]. These figures clearly indicate that CSF concentrations in the healthy rat correlate rather well with brain ECF concentrations although there is a trend for the Kpuu, CSF values to be slightly larger than the Kpuu, brain values. This may, at least in part, be due to more efficient drug elimination from brain ECF by active efflux mechanisms at the BBB, like mediated by *P*-glycoprotein (Pgp) and breast cancer resistance protein (BCRP) [56].

The relationship between rat and human Kpuu, CSF is more variable. There is a trend for human Kpuu, CSF to be larger than rat Kpuu, CSF. One reason for this may be that human CSF most probably has been obtained under disease conditions. These conditions might have influenced blood–brain transport, blood flow and/or metabolism, therewith affecting the Kpuu values [43, 57, 58]. For moxalactam there is some proof for this as one of the two human values for Kpuu, CSF has been obtained under “normal” conditions, and the other under “diseased” conditions (Fig. 4b), [55]. Here, the “diseased” Kpuu, CSF was larger than the “healthy” Kpuu, CSF value. It may may very well be that this Kpuu, CSF has reflected the corresponding Kpuu, brain in humans, as is known for moxifloxacin in bacterial meningitis [59]. In addition to the possibility of disease influencing Kpuu, CSF, other factors such as the site of CSF withdrawal and age of the patients may play a role [58, 60].

There is one example of a study in which brain ECF, brain tissue, CSF, and serum concentrations of antiepileptic drugs were obtained intraoperatively from patients with intractable epilepsy [45], and compared. Specifically, carbamazepine (CMZ), 10-hydroxy-carbazepine (10-OH-CZ, the active metabolite of oxcarbazepine), lamotrigine (LTG), and levetiracetam (LEV) were investigated. Overall it was found that CSF concentrations were significantly higher than corresponding brain ECF concentrations. This ratio was ~2.5 for CMZ, ~3.5 for both 10-OH-CZ and LEV, and ~4.0 for LMT. This is extremely valuable information, as this relationship has been shown for the first time in human. For rats, under control conditions, the CSF/brain ECF ratio for CMZ was ~0.7 for two independent studies [56, 61] and ~1.5 for LMT [62].

Overall, it can be concluded that CSF drug concentrations in rat, under controlled conditions, can give a rather good indication, but not a reliable prediction of brain ECF concentrations [3]. When it comes to humans, the conditions of the human individuals are highly variable, such that the relationship between rat CSF and human CSF concentrations gets more scattered. But, apart from the only study by Rambeck et al. [45], information about the relation between human CSF and human brain ECF concentrations is still unavailable.

As human CSF is the only fluid that can be obtained from human CNS, it is for that reason highly valuable and new approaches to derive more information from human CSF data are needed. To that end, much more mechanistic insight is needed on the processes that govern intrabrain distribution of drugs. This can be obtained from animal studies, and combined with data on the relationship between CSF and brain ECF concentrations from those exceptional cases where microdialysis is allowed to be used in individual patients.

**Fig. 5:**
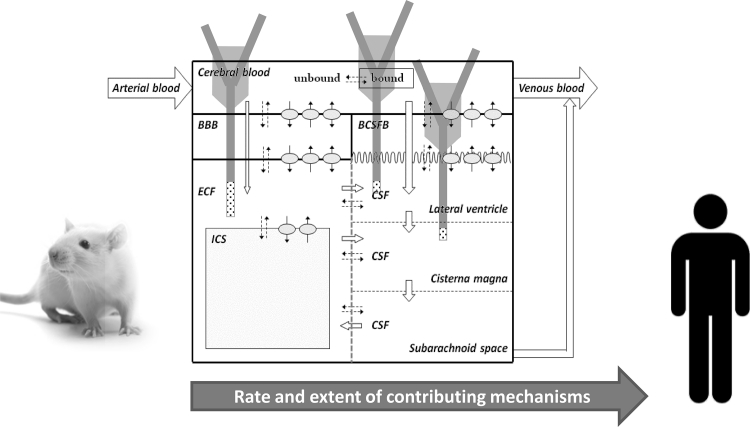
Using the parallel microdialysis probe design, pharmacokinetics of drugs at multiple sites in the brain can be assessed in individual animals, allowing direct comparison of concentration–time profiles of the free drug at these sites

**Fig. 6:**
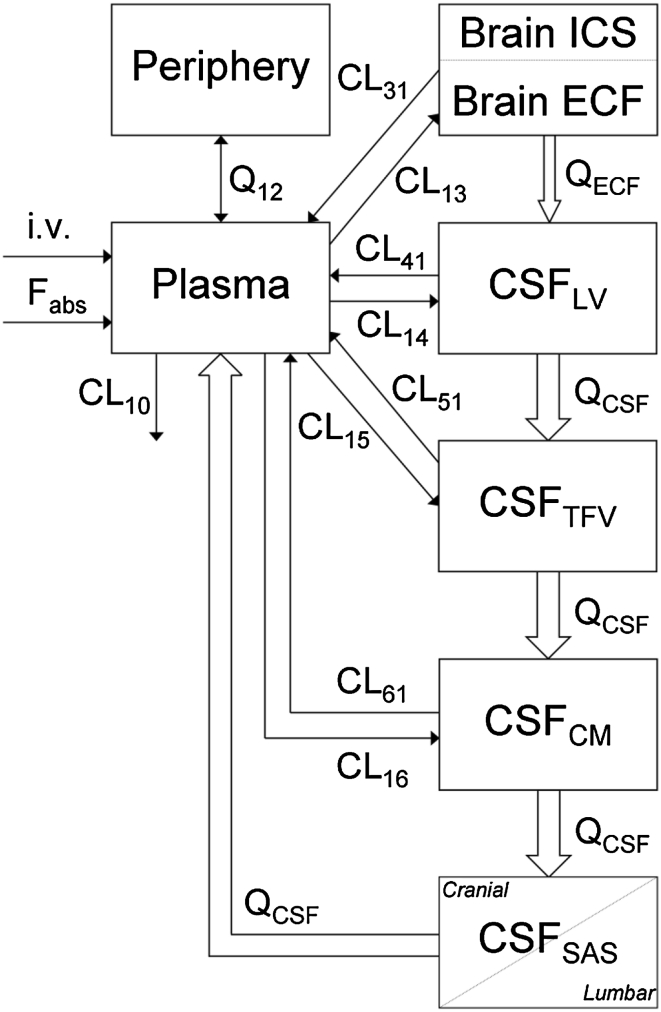
Physiologically based pharmacokinetic model of brain distribution, as derived for acetaminophen in the rat in which *plasma*, *brain ECF*, *CSF* in lateral ventricle, and *CSF* in cistern magna were assessed in parallel

**Fig. 7:**
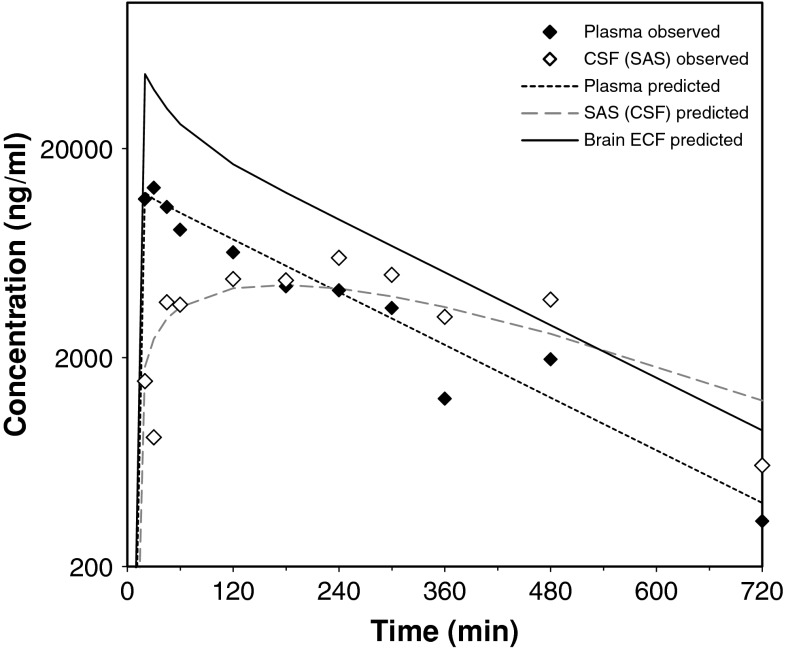
Successfull prediction of unbound human plasma and lumbal CSF (subarachnoid space, SAS), obtained by Bannwarth et al. [60], by the preclinically derived PBPK model for acetaminophen, and prediction of human brain ECF pharmacokinetics (Westerhout et al. [25])

Recently, Westerhout et al. [25] performed a series of studies using a multiple microdialysis probe design in the rat for measuring brain ECF concentrations and concomitant CSF concentrations in the lateral ventricle and cisterna magna (Fig. 5). Acetaminophen was the first paradigm compound, representing a moderately lipophilic drug for which only passive transport across the blood–brain barriers and within the brain occurs. The data obtained were used to build an advanced semi-physiological mathematical model of the brain, in which literature values of rat brain physiology were incorporated (Fig. 6), This model was able to describe the rat pharmacokinetic data in the different brain compartments adequately. Interestingly, after scaling the physiological parameters to the human values, this model was able to predict available literature data on human lumbar CSF concentrations [63]. This gave much confidence in the model structure and values, as well as in the prediction of brain ECF acetaminophen concentrations in human (Fig. 7). Recently, studies have been performed with quinidine, as a moderately lipophilic Pgp substrate. The Pgp inhibitor tariquidar was used to learn more about the impact of specifically Pgp functionality [REF, manuscript under revision, just accepted for publication in JPKPD if revised]. Furthermore, studies have been performed for methotrexate, as a hydrophilic Mrp substrate, in which probenecid was used to study the impact of MRP functionalities on brain distribution (yet unpublished data).

## Conclusions and perspectives

The development of drugs for CNS disorders has encountered high failure rates. In part this has been due to the sole focus on BBB permeability of drugs, without taking into account all other processes that determine drug concentrations at the target site. Moreover, conditional dependence of these processes has typically been neglected. The difficulty therefore relies in how to predict human brain target site concentrations, which in many cases most closely relates to brain ECF concentrations. In human, the only fluid that can be obtained is CSF.

So far it has been shown that in animals, under presumed steady state conditions, CSF concentrations tend to overestimate brain ECF concentrations. No clear relationship to the physicochemical properties of the drug could be identified [27]. However, active efflux transport at the brain ECF level for Pgp substrates seems to contribute to this phenomenon [16, 55, 56]. When comparing rat CFS concentrations with human CSF concentrations, human CSF concentrations tend to be higher and display much more variability. It should also be considered that the comparison here is made between *healthy* rats and humans that most probably beared a (CNS) disease that invalidates any direct comparison with healthy rats.

In order to be able to more accurately predict human brain ECF concentrations, our understanding of CNS complexity in terms of intrabrain pharmacokinetic relationships, brain distribution, and the influence of CNS disorders needs to increase. This could be achieved by performing more studies to obtain pharmacokinetic data in parallel from brain ECF and CSF compartments in animals, and building a generic PBPK model of brain distribution [11, 25, 29, 64]. Such a model should include influences of drug properties, fluid flows, transporter functionalities and the influence of different disease conditions. Finally, the free concentrations in different sites of the brain should be connected to measures of target site engagement and CNS effects to learn from best predictor concentrations for particular targets.

CSF fluid is the only accessible fluid in the human CNS, and for that reason is of high value. If the rat PBPK model is scaled to humans and the model successfully predicts human CSF concentration in the compartment where the information was obtained from, this increases our trust in the prediction of brain ECF concentrations using the model [25, 89]. The model could also be refined by including more “generating data—model prediction—model validation—confirm or adjust” cycles.

In summary, human CSF data can be of high value if we have the knowledge that enables us to extrapolate the CSF concentrations to brain target site concentrations, in order to predict the CNS effect in individual human conditions.

## References

[1] Hurko O, Ryan JL (2005). Translational research in central nervous system drug discovery. Neurotx.

[2] De Lange ECM, Ravenstijn PGM, Groenendaal D, van Steeg TS (2005). Toward the prediction of CNS drug effect profiles in physiological and pathological conditions using microdialysis and mechanism-based pharmacokinetic-pharmacodynamic modeling. AAPS J.

[3] Lin JH (2008). CSF as a surrogate for assessing CNS exposure: an industrial perspective. Curr Drug Metab.

[4] Hammarlund-Udenaes M, Bredberg U, Fridén M (2009). Methodologies to assess brain drug delivery in lead optimization. Curr Topics Med Chem.

[5] Read KD, Braggio S (2010). Assessing brain free fraction in early drug discovery. Exp Opin Drug Metab Toxicol.

[6] Watson J, Wright S, Lucas A, Clarke KL, Viggers J, Cheetham S, Jeffrey P, Porter R, Read KD (2009). Receptor occupancy and brain free fraction. Drug Metab Dispos.

[7] Jeffrey Ph, Summerfield S (2010). Assessment of the blood-brain barrier in CNS drug discovery. Neurobiol Dis.

[8] Liu X, Smith BJ, Chen C, Callegari E, Becker SL, Chen X, Cianfrogna J, Doran AC, Doran SD, Gibbs JP, Hosea N, Liu J, Nelson FR, Szewc MA, Van Deusen J (2005). Use of a physiologically based pharmacokinetic model to study the time to reach brain equilibrium: an experimental analysis of the role of blood-brain barrier permeability, plasma protein binding, and brain tissue binding. J Pharmacol Exp Ther.

[9] Hammarlund-Udenaes M (2010). Active-site concentrations of chemicals—are they a better predictor of effect than plasma/organ/tissue concentrations?. Basic Clin Pharmacol Toxicol.

[10] Bundgaard C, Sveigaard C, Brennum LT, Stensbøl TB (2012). Associating in vitro target binding and in vivo CNS occupancy of serotonin reuptake inhibitors in rats: the role of free drug concentrations. Xenobiotica.

[11] Westerhout J, Danhof M, de Lange ECM (2011). Preclinical prediction of human brain target site concentrations: considerations in extrapolating to the clinical setting. J Pharm Sci.

[12] De Lange ECM, Zurcher C, Danhof M, De Boer AG, Breimer DD (1995). Repeated microdialysis perfusions: periprobe tissue reactions and BBB permeability. Brain Res.

[13] Spector R, Spector AZ, Snodgrass SR (1977). Model for transport in the central nervous system. Am J Physiol.

[14] Begley (2000). In: The blood-brain barrier and drug delivery to the CNS, 2000

[15] Abbott NJ (2004). Evidence of bulk flow of brain interstitial fluid: significance for physiology and pathology. Neurochem Int.

[16] De Lange ECM (2004). Potential role of ABC transporters as a detoxification system at the blood-cerebrospinal fluid-barrier. Adv Drug Del Rev.

[17] Ghersi-Egea JF, Strazielle N, Murat A, Jouvet A, Buénerd A, Belin MF (2006). Brain protection at the blood-cerebrospinal fluid interface involves a glutathione-dependent metabolic barrier mechanism. J Cereb Blood Flow Metab.

[18] Ghersi-Egea JF, Mönkkönen KS, Schmitt C, Honnorat J, Fèvre-Montange M, Strazielle N (2009). Blood-brain interfaces and cerebral drug bioavailability. Rev Neurol (Paris).

[19] Johanson CE, Duncan JA, Klinge PM, Brinker T, Stopa EG, Silverberg GD (2008). Multiplicity of cerebrospinal fluid functions: new challenges in health and disease. Cerebrospinal Fluid Res.

[20] Vladić A, Klarica M, Bulat M (2009). Dynamics of distribution of 3H-inulin between the cerebrospinal fluid compartments. Brain Res.

[21] Iliff JJ, Wang M, Liao Y, Plogg BA, Peng W, Gundersen GA, Benveniste H, Vates GE, Deane R, Goldman SA, Nagelhus EA, Nedergaard M (2012). A paravascular pathway facilitates CSF flow through the brain parenchyma and the clearance of interstitial solutes, including amyloid β. Sci Transl Med.

[22] Brown PD, Davies SL, Speake T, Millar ID (2004). Molecular mechanisms of cerebrospinal fluid production. Neuroscience.

[23] Sharma HS, Johanson CE (2007). Blood–cerebrospinal fluid barrier in hyperthermia. Prog Brain Res.

[24] Biceroglu H, Albayram S, Ogullar S, Hasiloglu ZI, Selcuk H, Yuksel O, Karaaslan B, Yildiz C, Kiris A (2012). Direct venous spinal reabsorption of cerebrospinal fluid: a new concept with serial magnetic resonance cisternography in rabbits. J Neurosurg Spine.

[25] Westerhout J, Ploeger B, Smeets J, Danhof M, De Lange ECM (2012). Physiologically-based pharmacokinetic modeling to investigate regional brain distribution kinetics in rats. AAPS J.

[26] De Lange ECM, Danhof M (2002). Considerations in the use of cerebrospinal fluid pharmacokinetics to predict brain target concentrations in the clinical setting: implications of the barriers between blood and brain. Clin Pharmacokinet.

[27] Shen DD, Artru AA, Adkison KK (2004). Principles and applicability of CSF sampling for the assessment of CNS drug delivery and pharmacodynamics. Adv Drug Deliv Rev.

[28] Lee G, Bendayan R (2004). Functional expression and localization of *P*-glycoprotein in the central nervous system: relevance to the pathogenesis and treatment of neurological disorders. Pharm Res.

[29] De Lange, ECM (2013). The use of the mastermind research approach: factors in brain distribution and prediction of human brain target site kinetics and CNS drug effects. Fluid Barriers CNS (in press) 10.1186/2045-8118-10-12 PMC3602026 23432852

[30] Bonati M, Kanto J, Tognoni G (1982). Clinical pharmacokinetics of cerebrospinal fluid. Clin Pharmacokinet.

[31] Hammarlund-Udenaes M, Fridén M, Syvänen S, Gupta A (2008). On the rate and extent of drug delivery to the brain. Pharm Res.

[32] Danhof M, Levy G (1984). Kinetics of drug action in disease states. I. Effect of infusion rate on phenobarbital concentrations in serum, brain and cerebrospinal fluid of normal rats at onset of loss of righting reflex. J Pharmacol Exp Ther.

[33] Bruce J, Tamarkin L, Riedel C, Markey S, Oldfield E (1991). Sequential cerebrospinal fluid and plasma sampling in humans: 24-hour melatonin measurements in normal subjects and after peripheral sympathectomy. J Clin Endocrinol Metab.

[34] Miyakawa Y, Meyer JS, Ishihara N, Naritomi H, Nakai K, Hsu MC, Deshmukh VD (1977). Effect of cerebrospinal fluid removal on cerebral blood flow and metabolism in the baboon: influence of tyrosine infusion and cerebral embolism on cerebrospinal fluid pressure autoregulation. Stroke.

[35] Bungay PM, Morrison PF, Dedrick RL (1990). Steady-state theory for quantitative microdialysis of solutes and water in vivo and in vitro. Life Sci.

[36] Scheller D, Kolb J (1991). The internal reference technique in microdialysis: a practical approach to monitoring dialysis efficiency and to calculating tissue concentration from dialysate samples. J Neurosci Methods.

[37] Olson RJ, Justice JB (1993). Quantitative microdialysis under transient conditions. Anal Chem.

[38] Bengtsson J, Boström E, Hammarlund-Udenaes M (2008). The use of a deuterated calibrator for in vivo recovery estimations in microdialysis studies. J Pharm Sci.

[39] De Lange EC, de Bock G, Schinkel AH, de Boer AG, Breimer DD (1998). BBB transport and *P*-glycoprotein functionality using MDR1A (−/−) and wild-type mice. Total brain versus microdialysis concentration profiles of rhodamine-123. Pharm Res.

[40] Hillered L, Persson L, Nilsson P, Ronne-Engstrom E, Enblad P (2006). Continuous monitoring of cerebral metabolism in traumatic brain injury: a focus on cerebral microdialysis. Curr Opin Crit Care.

[41] Lindberger M, Tomson T, Wallstedt L, Stahle L (2001). Distribution of valproate to subdural cerebrospinal fluid, subcutaneous extracellular fluid, and plasma in humans a microdialysis study. Epilepsia.

[42] Bouw R, Ederoth P, Lundberg J, Ungerstedt U, Nordstrom CH, Hammarlund-Udenaes M (2001). Increased blood-brain barrier permeability of morphine in a patient with severe brain lesions as determined by microdialysis. Acta Anaesthesiol Scand.

[43] Ederoth P, Tunblad K, Bouw R, Lundberg CJ, Ungerstedt U, Nordström CH, Hammarlund-Udenaes M (2004). Blood-brain barrier transport of morphine in patients with severe brain trauma. Br J Clin Pharmacol.

[44] Scheyer RD, During MJ, Spencer DD, Cramer JA, Mattson RM (1994). Measurement of carbamazepine and carbamazepine epoxide in the human brain using in vivo microdialysis. Neurology.

[45] Rambeck B, Jurgens UH, May TW, Pannek HW, Behne F, Ebner A, Gorji A, Straub H, peckman E-J, Pollman-Eden B, Loscher W (2006). Comparison of brain extracellular fluid, brain tissue, cerebrospinal fluid, and serum concentrations of antiepileptic drugs measured intraoperatively in patients with intractable epilepsy. Epilepsia.

[46] Poeppl W, Zeitlinger M, Donath O, Wurm G, Müller M, Botha F, Illievich UM, Burgmann H (2012). Penetration of doripenem in human brain: an observational microdialysis study in patients with acute brain injury. Int J Antimicrob Agents.

[47] Tisdall MM, Smith M (2006). Cerebral microdialysis: research technique or clinical tool. Br J Anaesth.

[48] Helmy A, Carpenter KL, Hutchinson PJ (2007). Microdialysis in the human brain and its potential role in the development and clinical assessment of drugs. Curr Med Chem.

[49] Nau R, Sorgel F, Eiffert H (2010). Penetration of drugs through the blood-cerebrospinal fluid/blood-brain barrier for treatment of central nervous system infections. Clin Microbiol Rev.

[50] Wong SL, Van Belle K, Sawchuk RJ (1993). Distributional transport kinetics of zidovudine between plasma and brain extracellular fluid/cerebrospinal fluid in the rabbit: investigation of the inhibitory effect of probenecid utilizing microdialysis. J Pharmacol Exp Ther.

[51] Malhotra BK, Lemaire M, Sawchuk RJ (1994). Investigation of the distribution of EAB 515 to cortical ECF and CSF in freely moving rats utilizing microdialysis. Pharm Res.

[52] Yang Z, Huang Y, Gan G, Sawchuk RJ (2005). Microdialysis evaluation of the brain distribution of stavudine following intranasal and intravenous administration to rats. J Pharm Sci.

[53] Kakee A, Terasaki T, Sugiyama Y (1996). Brain efflux index as a novel method of analyzing efflux transport at the blood-brain barrier. J Pharmacol Exp Ther.

[54] Fridén M, Gupta A, Antonsson M, Bredberg U, Hammarlund-Udenaes M (2007). In vitro methods for estimating unbound drug concentrations in the brain interstitial and intracellular fluids. Drug Metab Dispos.

[55] Ichikawa H, Itoh K (2011). Blood-arachnoid barrier disruption in experimental rat meningitis detected using gadolinium-enhancement ratio imaging. Brain Res.

[56] Kodaira H, Kusuhara H, Fujita T, Ushiki J, Fuse E, Sugiyama Y (2011). Quantitative evaluation of the impact of active efflux by *P*-gp and Bcrp at the BBB on the predictability of the unbound concentrations of drugs in the brain using cerebrospinal fluid concentration as surrogate. J Pharmacol Exp Ther.

[57] Djukic M, Munz M, Sörgel F, Holzgrabe U, Eiffert H, Nau R (2012). Overton’s rule helps to estimate the penetration of anti-infectives into patients’ cerebrospinal fluid. Antimicrob Agents Chemother.

[58] Kanellakopoulou K, Pagoulatou A, Stroumpoulis K, Vafiadou M, Kranidioti H, Giamarellou H, Giamarellos-Bourboulis EJ (2008). Pharmacokinetics of moxifloxacin in non-inflamed cerebrospinal fluid of humans: implication for a bactericidal effect. J Antimicrob Chemother.

[59] Sandberg DI, Crandall KM, Koru-Sengul T, Padgett KR, Landrum J, Babino D, Petito CK, Solano J, Gonzalez-Brito M, Kuluz JW (2010). Pharmacokinetic analysis of etoposide distribution after administration directly into the fourth ventricle in a piglet model. J Neurooncol.

[60] Bannwarth B, Netter P, Lapicque F, Gillet P, Péré P, Boccard E, Royer RJ, Gaucher A (1992). Plasma and cerebrospinal fluid concentrations of paracetamol after a single intravenous dose of propacetamol. Br J Clin Pharmacol.

[61] Lui X, van Natta K, Yeo H, Vilenski O, Weller PE, Worboys PD, Monshouwer M (2009). Unbound drug concentrations in brain homogenate and cerebral spinal fluid at steady state as a surrogate for unbound concentrations in brain interstitial fluid. Drug Metab Dispos.

[62] Walker MC, Tong X, Perry H, Alavijeh MS, Patsalos PN (2000). Comparison of serum, cerebrospinal fluid and brain extracellular fluid pharmacokinetics of lamotrigine. Br J Pharmacol.

[63] Kielbasa W, Stratford RE (2012). Exploratory translational modeling approach in drug development to predict human brain pharmacokinetics and pharmacologically relevant clinical doses. Drug Metab Dispos.

[64] Kielbasa W, Kalvass JC, Stratford R (2009). Microdialysis evaluation of atomoxetine brain penetration and central nervous system pharmacokinetics in rats. Drug Metab Dispos.

[65] Bass NH, Lundborg P (1973). Postnatal development of bulk flow in the cerebrospinal fluid system of the albino rat: clearance of carboxyl-(14C)inulin after intrathecal infusion. Brain Res.

[66] Cserr HF (1965). Potassium exchange between cerebrospinal fluid, plasma, and brain. Am J Physiol.

[67] Cserr HF, Cooper DN, Suri PK, Patlak CS (1981). Efflux of radiolabeled polyethylene glycols and albumin from rat brain. Am J Physiol.

[68] Dobbing J, Sands J (1973). Quantitative growth and development of human brain. Arch Dis Child.

[69] Kimelberg HK (2004). Water homeostasis in the brain: basic concepts. Neuroscience.

[70] Ito H, Inoue K, Goto R, Kinomura S, Taki Y, Okada K, Sato K, Sato T, Kanno I, Fukuda H (2006). Database of normal human cerebral blood flow measured by SPECT: i. comparison between I-123-IMP, Tc-99 m-HMPAO, and Tc-99 m-ECD as referred with O-15 labeled water PET and voxel-based morphometry. Ann Nucl Med.

[71] Harashima H, Sawada Y, Sugiyama Y, Iga T, Hanano M (1985). Analysis of nonlinear tissue distribution of quinidine in rats by physiologically based pharmacokinetics. J Pharmacokinet Biopharm.

[72] Stahle L, Borg N (2000). Transport of alovudine (3′-fluorothymidine) into the brain and the cerebrospinal fluid of the rat, studied by microdialysis. Life Sci.

[73] Deguchi Y, Inabe K, Tomiyasu K, Nozawa K, Yamada S, Kimura R (1995). Study on brain interstitial fluid distribution and blood-brain barrier transport of baclofen in rats by microdialysis. Pharm Res.

[74] Jacobs S, McCully CL, Murphy RF, Bacher J, Balis FM, Fox E (2010). Extracellular fluid concentrations of cisplatin, carboplatin, and oxaliplatin in brain, muscle, and blood measured using microdialysis in nonhuman primates. Cancer Chemother Pharmacol.

[75] Matsushita H, Suzuki H, Sugiyama Y, Sawada Y, Iga T, Kawaguchi Y, Hanano M (1991). Facilitated transport of cefodizime into the rat central nervous system. J Pharmacol Exp Ther.

[76] Granero L, Santiago M, Cano J, Machado A, Peris JE (1995). Analysis of ceftriaxone and cefodizime distribution in cerebrospinal fluid of and cerebral extracellular space in awake rats by in vivo microdialysis. Antimicrob Agents Chemother.

[77] Venkatakrishnan K, Tseng E, Nelson FR, Rollema H, French JL, Kaplan IV, Horner WE, Gibbs MA (2007). Central nervous system pharmacokinetics of the Mdr1 *P*-glycoprotein substrate CP-615,003: intersite differences and implications for human receptor occupancy projections from cerebrospinal fluid exposures. Drug Metab Dispos.

[78] Au-Yeung SC, Rurak DW, Gruber N, Riggs KW (2006). A pharmacokinetic study of diphenhydramine transport across the blood-brain barrier in adult sheep: potential involvement of a carrier-mediated mechanism. Drug Metab Dispos.

[79] Ooie T, Suzuki H, Terasaki T, Sugiyama Y (1997). Kinetic evidence for active efflux transport across the blood-brain barrier of quinolone antibiotics. J Pharmacol Exp Ther.

[80] Stain-Texier F, Boschi G, Sandouk P, Scherrmann JM (1999). Elevated concentrations of morphine 6-beta-d-glucuronide in brain extracellular fluid despite low blood-brain barrier permeability. Br J Pharmacol.

[81] Wang X, Patsalos PN (2003). A comparison of central brain (cerebrospinal and extracellular fluids) and peripheral blood kinetics of phenytoin after intravenous phenytoin and fosphenytoin. Seizure.

[82] Deguchi Y, Nowaza K, Yamada S, Yokoyama Y, Kimura R (1997). Quantitative evaluation of brain distribution and blood-brain barrier efflux transport of probenecid in rats by microdialysis. Possible involvement of the monocarboxylic acid transport system. J Pharmacol Exp Ther.

[83] Van Amsterdam C, Lemaire M (1997). Pharmacokinetic profile of SDZ EAA 494 in blood, brain, and CSF using microdialysis. Eur J Pharm Sci.

[84] Yang Z, Brundage RC, Barbhaiya RH, Sawchuk RJ (1997). Microdialysis studies of the distribution of stavudine into the central nervous system in the freely-moving rat. Pharm Res.

[85] Wang X, Ratnaraj N, Patsalos PN (2004). The pharmacokinetic inter-relationship of tiagabine in blood, cerebrospinal fluid and brain extracellular fluid (frontal cortex and hippocampus). Seizure.

[86] Mano Y, Higuchi S, Kamimura H (2002). Investigation of the high partition of YM992, a novel antidepressant, in rat brain—in vitro and in vivo evidence for the high binding in brain and the high permeability at the BBB. Biopharm Drug Dispos.

[87] Wang Y, Sawchuk RJ (1995). Zidovudine transport in the rabbit brain during intravenous and intracerebroventricular infusion. J Pharm Sci.

[88] Fridén M, Winiwarter S, Jerndal G, Bengtsson O, Wan H, Bredberg U, Hammarlund-Udenaes M, Antonsson M (2009). Structure—brain exposure relationships in rat and human using a novel data set of unbound drug concentrations in brain interstitial and cerebrospinal fluids. J Med Chem.

